# Injuries by marine and freshwater stingrays: history, clinical aspects of the envenomations and current status of a neglected problem in Brazil

**DOI:** 10.1186/1678-9199-19-16

**Published:** 2013-07-29

**Authors:** Vidal Haddad Junior, João Luiz Costa Cardoso, Domingos Garrone Neto

**Affiliations:** 1 Department of Dermatology and Radioteraphy, Botucatu Medical School, São Paulo State University (UNESP – Univ Estadual Paulista), Botucatu, São Paulo State, Brazil; 2 Private Clinic, Ubatuba town, São Paulo State, Brazil; 3 Fishing Engineering Course, São Paulo State University (UNESP – Univ Estadual Paulista), Registro, São Paulo State, Brazil

**Keywords:** Sting, Freshwater stingrays, Marine stingrays, Venomous animals, Venomous fish

## Abstract

Stingrays are a group of rays – cartilaginous fish related to sharks – that have whiplike tails with barbed, usually venomous spines and are found around the world, especially the marine species. Despite recent reports of accidents involving these fish, they are not aggressive, reacting only when stepped on or improperly handled. Injuries by stingrays are seldom mentioned by historians, although they have always been present in riverine communities of inland waters and in South American coasts. Indeed, envenomations by stingrays are quite common in freshwater and marine fishing communities. Although having high morbidity, such injuries are neglected because they have low lethality and usually occur in remote areas, which favor the use of folk remedies. In the present review article, historical aspects of injuries caused by stingrays in Brazil and their distribution on the coast of São Paulo state and riverine communities of the North, Midwest and Southeast regions were studied. In addition, other aspects were analyzed such as clinical features, therapeutic methods, preventive measures and trends in occurrence of these accidents in the country, particularly in areas in which freshwater stingrays had not been previously registered, being introduced after breaching of natural barriers.

## Introduction

Stingrays are not aggressive by nature, they only react when people step on them or handle them improperly. Historically, injuries provoked by stingrays are rarely mentioned, although these fish have always been found in riverine communities of South America. “If the victim defecates himself, he is not a man. If he doesn’t, it wasn’t a stingray” said a Brazilian fisherman from the Midwest region referring to the excruciating pain caused by a stingray sting [1].

Envenomations caused by stingrays are relatively common in fishing communities either from the sea or rivers [2-8]. Such accidents have low lethality and high morbidity, and since they tend to occur in remote areas, they are usually neglected, unreported and treated with folk medicine [2-9]. The information available on this problem is based on active search for cases or incomplete medical records of health facilities from the northern region, with inherent restrictions concerning data collection methods [3,6,10-14].

**Figure 1:**
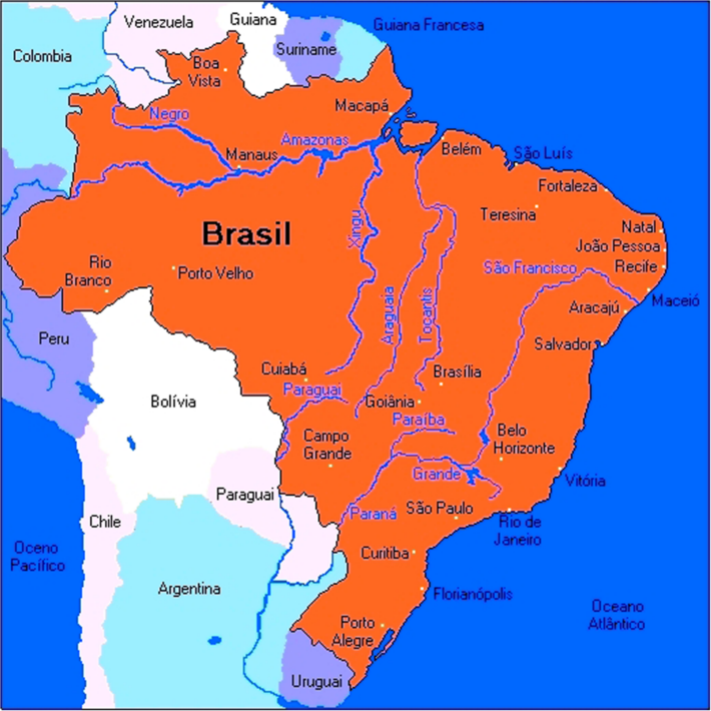
Map of Brazil in South America showing the Atlantic coast and the Amazon, Araguaia-Tocantins and Plata river basins.

Herein, historical aspects of injuries caused by stingrays in Brazil are commented. Their distribution is also studied; they are found throughout São Paulo state coast (marine stingrays are cosmopolitan) as well as in riverside communities of the North, Midwest and Southeast regions of the country (Figure 1). Additionally, we emphasized clinical aspects of stings, treatment, preventive measures and trends of occurrence of these accidents, especially in regions where freshwater stingrays had not been previously recorded.

## Review

### History: the early centuries

Since the discovery of Brazil there have been many reports describing stingrays and the injuries caused by these fish. The first author to mention them was André Thevet (*1503, †1592) in 1558. A Franciscan priest, he accompanied Villegagnon when France Antarctique was founded in Rio de Janeiro state [15]. Claude D’Abbeville (*?, †1616) was a Capuchin priest who was sent to Maranhão state for the colonial enterprise to found France Équinoxiale. In his book, published in 1614, is stated: “The jauebuíre has a long tail, in which center there is a dart shaped spine bigger than a finger, and whose injury is so dangerous that sometimes it is better to remove the affected limb” [15].

In his book *History of the animals and trees of Maranhão*[16], Frei Cristóvão de Lisboa (*1583, †1652) recorded 103 species of fish and published the first drawings of marine (*jabebura*) and freshwater (*jabepurapeni*) stingrays: “Jabebura […] is very strong and has spines on its back, after dried its skin serves as sand paper; they are so strong that neither an arrow nor a sword can pierce them, they have a spine on the tail that is very dangerous and their stings have already killed some people. […] Jabepurapeni is the freshwater ray, of a span and a half long, which can reach four and a half spans in diameter, they are very round, brown with red spots, their neck is covered with small spines and there are two long ones on the tail that are very dangerous and venomous; it is a very dangerous animal” [16].

In 1648, the physician Guillermo Piso (*1611, †1678) described the Dutch settlement in Brazil of Maurice of Nassau and also mentioned the marine stingrays: “Among all cartilaginous fish, stingrays are smooth and without stingers throughout the body, except for their end and tail, in which two short non-serrated arrow-shaped ones are seen, because of that our people named them pijlstart [in Dutch: arrow-tail] and the Indians, narinari. […] Deprived of stingers they are edible, as are almost all terrestrial or marine animals that only inoculate venom by stinging or biting. The afflicted limb is miserably affected by strong pain and sometimes paralysis” [15].

A collection of texts on fish and folklore brings a contribution of the naturalist and explorer Henry W. Bates (*1825, †1892): “[…] it is common stingrays in quiet bays and swimmers are often severely injured by them. The weapon of this fish is a robust blade with serrated edges and three inches long, which grows from the side of the fleshy long tail. I once saw a woman who was injured when bathing; she screamed ghastly, and had to be carried to the bed, where she stayed for a week in pain. I have seen strong men who were crippled for many months because of their stinger” [15,17].

### Epidemiology

#### Injuries by marine stingrays

**Figure 2:**
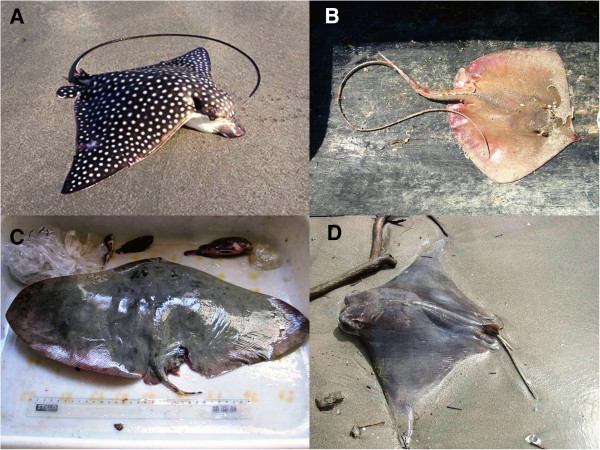
**Genera of marine stingrays. (A) ***Aetobatus narinari*, the spotted stingray; **(B)***Dasyatis guttata*, the whiptail ray; **(C)***Gymnura* sp., the butterfly or butter ray; **(D)***Rhinoptera bonasus*, the cownose stingray. Photos by: Ivan Sazima and Vidal Haddad Junior.

**Figure 3:**
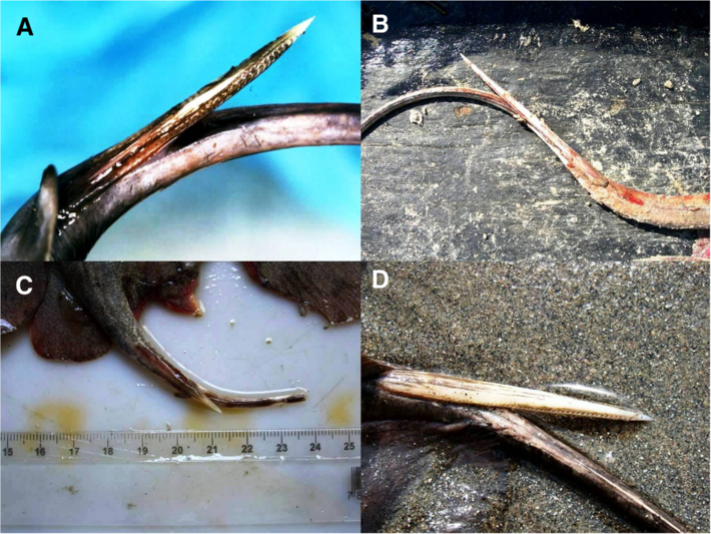
**Note the bony structures and serrated edges of stingers of the following species: (A) *Aetobatus narinari*; (B) *Dasyatis guttata*; (C) *Gymnura *sp.; and (D) *Rhinoptera bonasus*.** Photos by: Vidal Haddad Junior.

In São Paulo state, stingrays are usually found in coastal and estuarine environments. The four most common genera include *Aetobatus*, *Dasyatis*, *Gymnura* and *Rhinoptera* (Figure 2). As the name implies, stingrays have 1 to 4 stingers on the tail in the dorsal position that are used for self-defense (Figure 3). The stingers are covered by a toxin-producing glandular tissue; therefore, each sting causes intense inflammation, excruciating pain and skin necrosis (particularly in injuries provoked by freshwater stingrays).

*Aetobatus* and *Rhinoptera* stingrays are cosmopolitan and have benthic and pelagic habits. They are usually found in coastal waters near the surface and can leap out of the water and cluster in groups. Their stingers are located at the base of the tail, near the body, thereby minimizing its mobility and the severity of injuries. They spend much of their time moving through the water columns. On the northern coast of São Paulo, of 137 recorded accidents involving these fish, 80% were caused by stingrays of the *Rhinoptera* genus (cownose stingrays) [8]. Almost all injuries occurred when animals were accidentally captured in fishing nets or while shrimp trawling. Although rare, injuries caused by the spotted ray *Aetobatus narinari* also occur when these animals are unintentionally caught in fishing nets. The same applies to *Gymnura*, these benthic stingrays – also known as butterfly or butter rays – have excellent camouflage and rarely sting unless touched, since their tails are very short.

**Figure 4:**
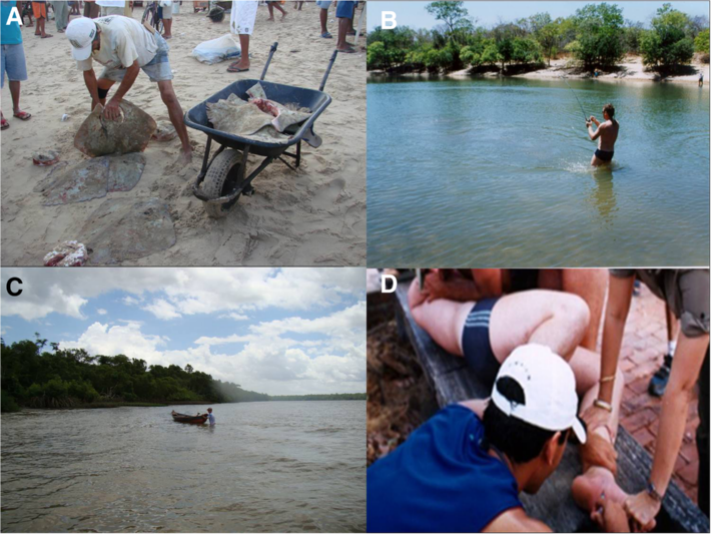
**Environments with high risk of injuries by stingrays. (A)** A fisherman handling marine stingrays in a fish market. **(B)** An amateur fisherman was fishing in a tributary of the Araguaia River (Midwest region of Brazil) when he was injured by a freshwater stingray. **(C)** A fisherman pushes his boat into shallow waters of a tributary of the Amazon River where rays are common: the risk of envenomation is high. **(D)** The pain of the man in **(B)** was excruciating. Photos by: Vidal Haddad Junior and Luís Fernando Beretta Gonçalves.

*Dasyatis* or whiptail stingrays also are globally distributed and can be observed in waters near coastal cities and also in shrimp trawls. These rays prefer shallow areas near mangroves and estuaries. In Ubatuba city, São Paulo state, injuries caused by *Dasyatis* affect mainly fishermen (Figure 4). Although incidents involving bathers and these stingrays are scarce, feet and ankles are the most affected limbs of people swimming in shallow waters on the Brazilian coast.

Fatalities with marine species of rays have not been recorded in Brazil. However, the literature and the media reported deaths in other countries due to thoracic and head wounds that had affected great blood vessels provoked by *Aetobatus narinari* stingrays jumping out of the water. Other dangerous situations related to these animals are rays captured in harpoons by divers and deaths of swimmers due to heart and lung injuries provoked by stings of rays during the practice of swimming or snorkeling [18-24].

#### Injuries caused by freshwater stingrays

**Amazon/Midwest regions** In Araguaia-Tocantins River Basin – mainly in the states of Tocantins, Mato Grosso and Pará – injuries caused by stingrays are more frequent in the dry season, especially during the months of July and August [6,25]. In this period beaches and sandbars are formed on the Araguaia River and its tributaries, and thousands of people look for recreational activities in such places [26,27]. Victims are usually stung on the lower limbs during the day, while swimming or moving through the water from a sandbar to another. Throughout the year accidents tend to decrease, but sporadic cases occur in professional and amateur fishermen. Among these people, accidents usually occur in the water, when animals are stepped on or when cast nets are used, because in this technique the fisherman sometimes stay in the water to collect the fishing net. Fish unloading or maintenance of boats (Figure 4C) may also result in accidents in the North and Midwest regions of the country.

Amateur fishermen are frequently hurt in April and May, when the fishing season is open for native species and people from the Southeast region look for the Amazonian and Midwestern rivers to practice sport fishing. On these occasions, stings are the result of manipulating animals accidentally caught in hooks or treading on camouflaged stingrays on beaches or sandbars (Figure 4). About twenty species of freshwater stingrays are considered valid nowadays, of which at least four can be found in the Araguaia-Tocantins Basin: *Potamotrygon henlei*, *Potamotrygon orbignyi*, *Potamotrygon scobina* and *Paratrygon aiereba*[28,29]. The latter is very unlikely to cause harm, because of its short tail and the position of the stingers. *Potamotrygon* rays have robust tails with stingers in mid-distal position that frequently cause accidents.

In the middle course of the Araguaia River (where the main fishing resorts are located), *Potamotrygon orbignyi* is usually the main species observed. In the same region, *Potamotrygon henlei* is habitually spotted near rocks. This species and *Paratrygon aiereba* tend to approach the margins during the night, which requires caution.

In the Tocantins River, due to flooding of extensive areas for hydroelectric plants, artificial islands and beaches were built and they are sought all over the year by local inhabitants for recreational activities. Thus, injuries tend to occur throughout the year. It is also noteworthy that in impoundment areas some environmental conditions such as the reduction of stream flow and the proliferation of food items included in the diet of rays may lead to population increase and, therefore, the interaction with humans, as it has been observed in areas surrounding Tucuruí lake (Marabá and Tucuruí, Pará state) and the Lajeado hydroelectric dam (Palmas, Tocantins state).

##### Southeast/Midwest regions

Although stingrays have been observed in the rivers of the Plata Basin since the colonization of South America, there are still many taxonomic uncertainties since these initial reports of the species in the Paraná River, in Santa Fé Province, Argentina [30-40]. The last systematic review of the family Potamotrygonidae and the most current listing of potamotrigonids considered valid suggest the existence of at least five species of rays in the Paraná-Paraguay Basin: *Potamotrygon brachyura*, *P. falkneri*, *P. histrix*, *P. motoro* and *P. schuhmacheri*[28,29,41].

**Figure 5:**
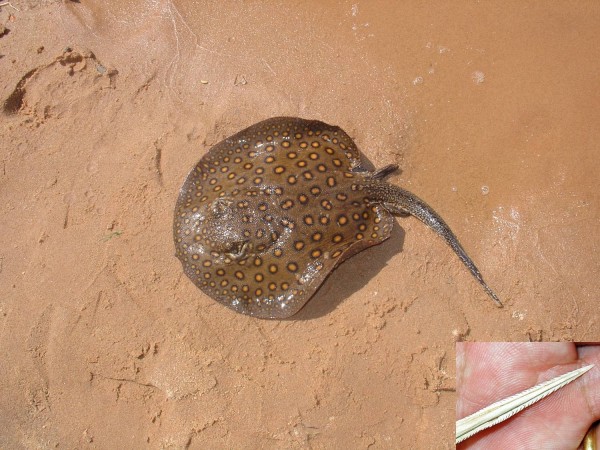
***Potamotrygon motoro*, the most common species of freshwater stingray.** This ray is found in most regions of Brazil. Photo by: Vidal Haddad Junior.

**Figure 6:**
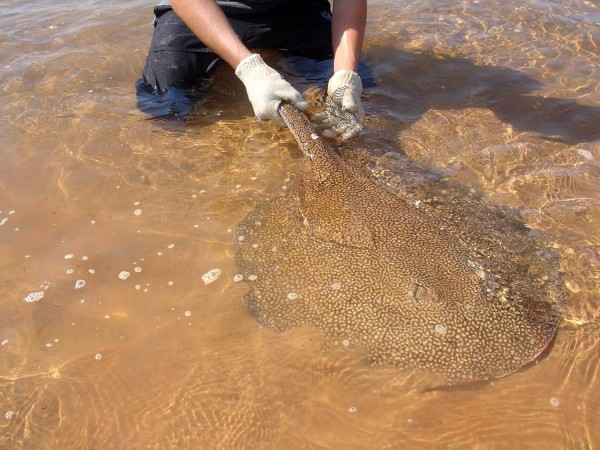
***Potamotrygon falkneri*, a species common in Paraná River, in Southeastern and Southern Brazil, Uruguay, Paraguay and Argentina.** Photo by: Vidal Haddad Junior.

In the Paraguay River Basin – as in the rivers of the North and Midwest regions of Brazil –freshwater stingray envenomations are part of daily lives of indigenous and riverine communities. However, accidents occur also in professional fishermen and river dwellers, wounded in the lower limbs during the execution of services in ports or recreational activities along rivers. Children can also be hurt in leisure activities, especially when they step on stingrays in the rivers. In amateur fishermen, injuries are mainly caused by improper handling of animals accidentally captured in hooks. There are also reports of accidents involving tourists and animals including horses and cattle, while bathing in river beaches or crossing rivers. In a study conducted in the region of Corumbá town, Mato Grosso do Sul state, the period in which the accidents were more frequent was found to be during the austral winter, between July and September [6]. Of the 18 cases investigated, 90% occurred during the day and the victims were males, aged between 29 and 46 years. The species *P. motoro*, of the Potamotrygonidae family, was the most common in the research site, which suggests that it was involved in most cases (Figure 5). *Potamotrygon falkneri* (Figure 6) is also found in the Paraguay River Basin and is related to human injuries as well [42].

In the Paraná River Basin, the fear of injuries by freshwater stingrays and other aquatic animals has been present for centuries, as evidenced by the reports of Sergeant José Teotônio Juzarte who in 1769 wrote: “[…] The waters of Paraná River are sometimes red and pestilential, and lashings of stingrays and scissors-toothed fish keep men screaming all day” [40]. Mariano Castex, a missionary living in Santa Fé, Argentina, carried out studies on various aspects related to freshwater stingrays, especially about their taxonomy, action of toxins and injuries [30-38].

In the Brazilian stretch of the Paraná River, stingray envenomations appeared over the past 20 years. An important natural barrier – the Seven Falls of Guaíra (Paraná state) – served as a natural barrier preventing species of the Upper Paraná River to colonize other regions. However, with the creation of Itaipu hydroelectric dam in 1982, these falls were submerged, allowing the movement of several fish species upstream, including rays that are taking advantage of locks installed at the dam to expand their distribution area [1,12].

Despite taxonomic uncertainties, studies in the region demonstrated that at least three species of potamotrigonids settled, to a greater or lesser extent, in invaded areas: *Potamotrygon falkneri*, *P. motoro* and *P. schuhmacheri*[7,10,12,13,43]. The first two are the most abundant species of the Upper Paraná River (Figures 5 and 6), and are found in the Itaipu Lake, in the lower courses of Tietê and Paranapanema rivers and other tributaries, more than 300 km above its threshold dispersion [7,10,12,13].

In the upper course of the Paraná River, injuries are reported by inhabitants and also tourists, who are often unaware of the presence of these animals in the area. Epidemiological surveys and ethno-ecological studies involving fishermen and inhabitants of Guaíra town (Paraná state), Presidente Epitácio town (São Paulo state), Novo Porto XV (Mato Grosso do Sul state), Castilho town (São Paulo state), Três Lagoas town (Mato Grosso do Sul state) and Itapura town (São Paulo state) revealed that freshwater stingray migration to that was more evident in the past decade and that envenomations were a sign of its presence [1,6,7,11,12].

The survey also showed lack of information about the fish and procedures to be followed in case of injuries. A respondent said: “Stingray meat is bad because of the venom, so we kill them. They can only be used to make medicines” (VHJ, pers. obs.). Additionally, health professionals often demonstrate lack of knowledge about the action of the venom and/or correct procedures to be followed in case of an accident.

Such situation is alarming because rays are reaching densely populated areas, where most inhabitants are unaware of how to prevent accidents and to treat wounds they may cause. If we take into account the existence of numerous properties by rivers, hotels and intense practice of fishing activity in the region of Upper Paraná River, it is expected that the negative interaction between humans and stingrays will be more intense, causing important changes in epidemiological profiles of envenomations in southeastern Brazil. Thus, we emphasize the importance of educational activities among the population and the training of health professionals near risk areas, as well as monitoring of the invasion process and reporting of accidents.

### Clinical aspects of envenomations

Pain and tissue necrosis at the sting site are constant and emphasized by several authors since the first reports of the injuries by freshwater stingrays. Vellard in 1931 described the clinical manifestations of stingray envenomations: “The rays often sting on the feet or lower third of the leg. The wounds are deep and wide; torn tissue is already painful, independent of the venom action. Only a few minutes later, five to ten or even more, symptoms start to manifest. Initially, there are very strong cramps extending to the base of the injured limb, which sometimes are generalized. The injured limb is immobilized in flexion and painful contractures begin; suffering becomes intolerable and the patient cannot stop screaming. The pain persists in the same intensity for ten or twenty hours and then slowly attenuates, but only disappears a few days later. Shortly after the accident, the injured area begins to burn, turns hot and red, and the sensation is often accompanied by blood suffusion. In general these wounds are poorly cared, which leads to lymphangitis and adenitis. Later, the necrotic tissues are detached due to ulcers, causing in many cases spontaneous amputation of toes, feet or hands. Healing always takes a long period. Besides the pain, general symptoms appear to be, generally, unimportant” [44]. Mello-Leitão [45], in 1948, reproduced the text above in his book.

In 1934, Couto de Magalhães [46] observed that “[…] the resulting wound (of the sting) is a problem, since the irregularity of the cut – because the stinger leaves a viscous product – greatly contributes to complicate the injury. There is more fear among people of the crew of these fish than snakes”. Castex [34] defined what he called the “enfermedad paratrygónica” after watching dozens of cases in Argentina: “The onset is sudden, with tearing sharp acute and violent pain with or without cramps, anxiety and restlessness and little or no general symptoms”. During this state or period there are “malaise (not constant), anxiety and restlessness (not constant), hyperthermia discrete (non-constant), pain, red moderate and localized edema, eschar formation constant and segregating perilesional and ulcer torpid”*.* The disease evolves during “20 days to several months” and the complications can be “traumatic with superimposed infection, gangrene, fistula, neurotrophic disorders, hypersensitization to various allergens and ruptured blood vessels by the ulcerous process” [35].

In our observations, based on a total of more than 400 accidents reported along the Brazilian coast and rivers of the Plata Basin (Paraná-Paraguay rivers in São Paulo, Paraná and Mato Grosso do Sul states) and Tocantins-Araguaia Basin (Goiás, Mato Grosso and Tocantins states), the injuries were provoked by the stingrays of Dasyatidae and Rhinopteridae families (marine rays) and Potamotrygonidae (freshwater stingrays). Although Dasyatidae rays are frequently involved in accidents, *Rhinoptera bonasus* was predominant in the research area (north coast of São Paulo state), followed by *Dasyatis guttata*. One hundred and thirty-seven accidents by marine rays were recorded. Most accidents by freshwater stingrays (a total of 275) were caused by the species *P. motoro* and *Potamotrygon falkneri* found in Plata Basin. About 60% of the victims were walking in the water or catching baits for fishing among macrophytes or providing small services in ports, while most of the victims of marine rays were fishermen during their practice. Over 90% of patients had stings in lower limbs and less than 10% in the hands in both marine and inland environments.

**Figure 7:**
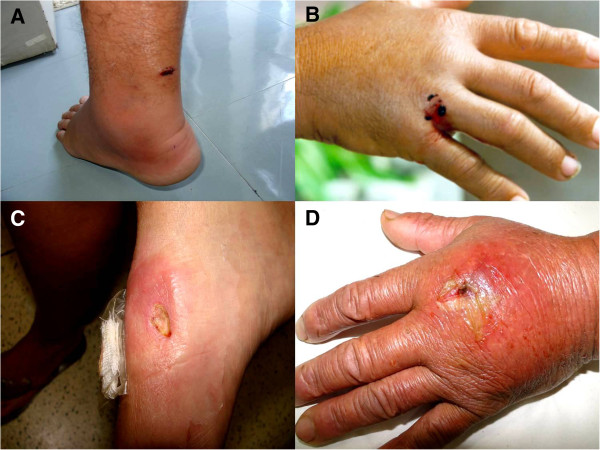
**Envenomations caused by marine stingrays. (A)** Erythema and edema in a heel injury. **(B)** Fisherman hand injured by a stingray. **(C)** Ulcer on the left foot of a fisherman. **(D)** Hand of a fisherman stung by a ray, the wound was associated with bacterial infection. This kind of injury is common in fishermen of shrimp communities, since the caught stingrays remain at the bottom of the boat. Photo by: Vidal Haddad Junior.

**Figure 8:**
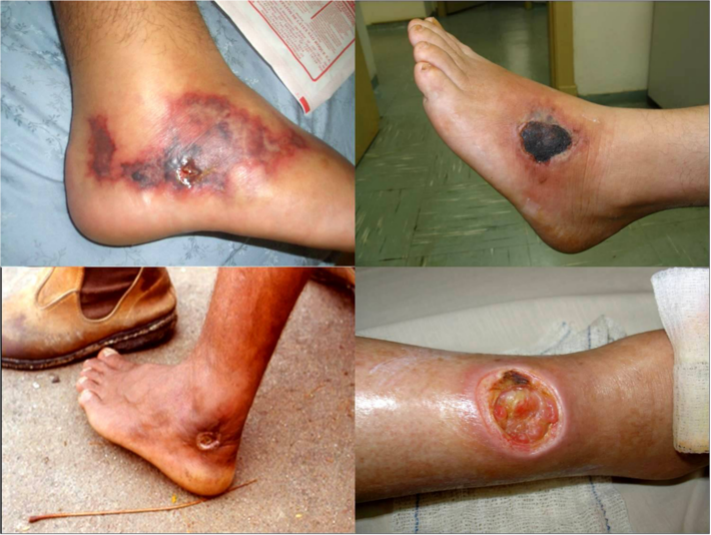
**Extensive necrosis on the lower limbs of amateur and professional fishermen of the central regions of Brazil.** Envenomations by freshwater stingrays tend to cause skin necrosis and chronic ulcers in the victim, who in most cases had stepped on the stingray in shallow waters. Photo by: Vidal Haddad Junior.

Severe pain was the main symptom, appearing immediately after the sting and causing behavioral changes in patients due to its intensity. Local edema and erythema, as well as cutaneous necrosis of varying intensity were also present (Figure 7). Freshwater stingray injuries are more severe than those caused by marine animals, considering skin necrosis as a parameter, since the pain is intense in both accidents (Figure 8). The rate of skin necrosis in marine ray wounds was about 75%, but it was near 100% in envenomations caused by freshwater stingrays [8].

Haddad Jr. et al. [6] and Barbaro et al. [47], comparing tissue samples from stings of *Dasyatis guttata* and *Potamotrygon falkneri* demonstrated that the skin necrosis observed in laboratory animals were more intense in mice injected with extracts derived from freshwater stingrays than those from marine rays as prevously observed during the assistance of stingray victims. Lethal, dermonecrotic and myotoxic activities were only detected in tissue extracts of *P. falkneri* stungs while edematous action was dose-dependent and similar in both extracts. The presence of coagulant action, direct hemolysis and phospholipase A2 was not detected. Both extracts showed caseinolytic, gelatinolytic and fibrinogenolytic activities, which were not caused by the action of metalloproteinases. Hyaluronidase activity was detected in extracts of *P. falkneri* and the presence of cross-reaction, tested using rabbit antibodies was demonstrated by ELISA and Western blotting. Although nociceptive action has been observed in both extracts, *P. falkneri* showed an activity two times higher than *D. guttata*, suggesting higher toxicity. Although clinical manifestations are clearly more intense in accidents caused by freshwater rays, the difference between the nociceptive activity observed in two groups need to be further investigated, because the thermolability of toxins that cause pain is closed related to environmental conditions.

The pattern of the resulting ulcer may be characteristic: in large necrosis, we observed that, from the central point where the stinger perforated the sting, new circular areas of necrosis are formed, resembling concentric circles caused by a stone thrown into the water or the surface of an onion cut longitudinally (Figure 6). These ulcers that look like “a stone in the water” or “onion cut” do not appear in all accidents, but when present, they help in clinical diagnosis. The healing of the ulcer is reached after months of evolution, leaving large scars on compromised areas.

The systemic symptoms observed are usually fever, cold sweats, nausea, vomiting and restlessness, associated to the pain and stress of the victims. The acute manifestations (within 24 hours) are extremely disabling, demonstrating the importance of such envenomations from the point of view of occupational health. The healing period of ulcers was about three months, but it was not possible to evaluate the rate of secondary infection involved in the process.

### Treatment

The treatment of the injuries involving freshwater stingrays is accompanied by a huge variety of folk remedies, superstitions and legends. Among other measures, the Brazilian indigenous people and riverbank inhabitants recommend application of human urine on the affected spot, the rubbing of catfish eyes or even – as is common in the Araguaia and Paraguai rivers and their tributaries – the placement of the stung area in contact with human female external genitalia (!), considered infallible procedure for the pain control. Of course, not all recommendations are acceptable from the standpoint of science.

In 1965, Castex in the communication “enfermedad paratrygónica”, presented at the VIII International Congress of Internal Medicine in Argentina, proposed measures for the treatment of stingray envenomation [37]. The immediate goal was the pain control and the prevention of skin necrosis. Therefore, the author suggested intensive washing of the wound and early removal of the affected area in an attempt to eliminate the venom, the use of local (novacaine) or systemic analgesics (opiates) for pain control, antibiotics at the first sign of infection and administration of sodium thiosulphate and calcium gluconate intravenously to prevent the chronicity of the injury. In the treatment of chronic lesions, the measures were rest, the use of corticosteroids and cleaning the ulcer with saline and hydrogen peroxide. The author mentioned the temporary incapacitation of the patient, who should be in bed for 60 days, and the absolute inability in the first fifteen days, which demonstrates the great potential morbidity of the accident.

Currently, there is a routine for the care of patients that were injured by stingrays. It is recommended the immersion of the affected limb in hot water – not scalding (about 60°C) due to the properties of some labile toxins of the stingray venom and the vasodilatation caused by the hot temperature, since the venom promotes intense vasoconstriction and ischemia [1,6]. Pain usually decreases with this measure.

Other procedures include major surgical exploration of the fragments, intensive cleaning of lacerated wounds, tetanus prophylaxis, use of antibiotics, use of anesthetics on the affected area and systemic analgesics, including opioids, if the pain control is not achieved in the first two hours. Early excision of the affected area is recommended by some authors, but its application is not always possible and there is the problem of inaccurate delineation of the necrotic area in the early stages of the condition. Complications of chronic phase are treated with measures used for the healing of chronic ulcers: rest, intensive washing with soap and water and topical antibiotics. The use of corticosteroids is controversial since these drugs can increase the time required for the healing of the ulcer.

There is not a definitive therapy that is really effective for injuries by stingrays. Inactivation of toxins is the only ideal way to neutralize the consequences of the sting. To use such tool, it would be necessary to produce a serum against the venom. It is known that the envenomation is severe, disabling and potentially fatal if infected, but there is no statistical justification for the production of serum, due to the small number of reported injuries. It should be borne in mind, however, that the discussion about the production of serum becomes more valid when observing the symptoms of a victim recently stung and when one has in mind that the data obtained by reporting these accidents are virtually nonexistent.

### Prevention

The occurrence of injuries by stingrays, as well as other accidents caused by venomous animals, can be greatly minimized through the implementation of educational programs in the populations at risk. Pamphlets combined with lectures and workshops, as well as the dissemination of information through the media, posters and warning signs are measures that can be effective in preventing and reducing the number of accidents by rays in different parts of Brazil and the world.

Stingrays have predominantly benthic habits associated with seafloor and freshwater environments, where their camouflage often makes them virtually invisible, especially when the water has low visibility. Therefore, the recommendation for those who need to walk in the water in places known to be inhabited by rays, is that drag their feet or use a stick or paddle to grope the substrate, since this method blows away any rays in the path of the swimmer or fisherman.

In situations in which it is necessary to handle rays, such as when they are caught in hooks or fishing nets, it is recommended to immobilize their tail, at the level of the spines before handling. For this, one can use pliers, bits of wood, fishing boxes or similar objects, taking care not to injure the animal. To step on their tails, even when protected by rubber boots, is not recommended.

Divers and freedivers should be careful when swimming near the substrate, coral reefs and submerged plant, since rays are commonly hidden or even foraging at these sites and may act with hostility due to the sudden encounter or any accidental contact. Prophylaxis is recommended for similar underwater hunters, so caution is required when one is lurking for prey at the bottom of rivers and seas.

It is necessary to emphasize that differently to what happens with the venom apparatus of the vast majority of other venomous animals such as snakes, scorpions and spiders, stingrays derived from ancestral animals that already used this tool to scare off predators and not to capture prey. This reinforces the concept that rays are not animals that usually attack humans, using their stingers only to self-defense.

## Conclusions

Stingrays are venomous fish that can provoke severe envenomations in some populations as fishermen, bathers and riverside inhabitants of marine and freshwater environments. The injuries cause intense local pain and cutaneous necrosis, complicated by secondary infections and retention of fragments of the stingers in the wound. The envenomation is poorly studied, once the injuries occur in victims that live in distant places and is treated with popular measures, preventing the notification, although questionnaires answered by fishermen and riverines point up to 25% of victims in populations who live near these fish. The actual treatment uses only symptomatic measures (such as hot water for pain control) and preventive measures are virtually nonexistent. We concluded that the knowledge obtained and presented here require practical application in these communities, because the most severe and common accident in the inland aquatic environments of Brazil is also the most neglected by public health institutions and by the population.

## Consent

Verbal informed consent was obtained from the patients for publication of the accompanying images in Figures 4, 7 and 8.

## Competing interests

The authors declare that they have no competing interests.

## Authors’ contributions

VHJ and DGN are researchers on the theme venomous fish and organized the manuscript. Moreover, they are both authors of the photographs. JLCC wrote about the historical aspects of the stingray envenomations and is part of a study group on stingrays. All authors read and approved the final manuscript.
